# Survey of programmatic experiences and challenges in delivery of hepatitis B and C testing in low- and middle-income countries

**DOI:** 10.1186/s12879-017-2767-0

**Published:** 2017-11-01

**Authors:** Azumi Ishizaki, Julie Bouscaillou, Niklas Luhmann, Stephanie Liu, Raissa Chua, Nick Walsh, Sarah Hess, Elena Ivanova, Teri Roberts, Philippa Easterbrook

**Affiliations:** 1 Global Hepatitis Programme, World Health Organization, 20 Avenue Appia, 1211, 27 Geneva, Switzerland; 2 Médecins du Monde, 62 rue Marcadet, 75018 Paris, France; 3 World Health Organization, Regional Office of the Western Pacific, United Nations Avenue, 1000 Manila, Philippines; 4 Foundation for Innovative New Diagnostics, Campus Biotech, Building B2, Level 0, 9 Chemin des Mines, 1202 Geneva, Switzerland

**Keywords:** Hepatitis testing, WHO guidelines on hepatitis B and C testing, Programme experience, Feasibility, Low- and middle-income countries

## Abstract

**Background:**

There have been few reports on programmatic experience of viral hepatitis testing and treatment in resource-limited settings. To inform the development of the 2017 World Health Organization (WHO) viral hepatitis testing guidance and in particular the feasibility of proposed recommendations, we undertook a survey across a range of organisations engaged with hepatitis testing in low- and middle-income countries (LMICs). Our objective was to describe current hepatitis B and C testing practices across a range of settings in different countries, as well as key barriers or challenges encountered and proposed solutions to promote testing scale-up.

**Methods:**

Hepatitis testing programmes in predominantly LMICs were identified from the WHO Global Hepatitis Programme contacts database and through WHO regional offices, and invited to participate. The survey comprised a six-part structured questionnaire: general programme information, description of hepatitis testing, treatment and care services, budget and funding, data on programme outcomes, and perceptions on key barriers encountered and strategies to address these.

**Results:**

We interviewed 22 viral hepatitis testing programmes from 19 different countries. Nine were from the African region; 6 from the Western Pacific; 4 from South-East Asia; and 3 from Eastern Europe. All but four of the programmes were based in LMICs, and 10 (45.5%) were supported by non-governmental or international organizations. All but two programmes undertook targeted testing of specific affected populations such as people living with HIV, people who inject drugs, sex workers, health care workers, and pregnant women. Only two programmes focussed on routine testing in the general population. The majority of programmes were testing in hospital-based or other health facilities, particularly HIV clinics, and community-based testing was limited. Nucleic acid testing (NAT) for confirmation of HCV and HBV viraemia was available in only 30% and 18% of programmes, respectively. Around a third of programmes required some patient co-payment for diagnosis. The most commonly identified challenges in scale-up of hepatitis testing were: limited community awareness about viral hepatitis; lack of facilities or services for hepatitis testing; no access to low cost treatment, particularly for HCV; absence of national guidance and policies; no dedicated budget for hepatitis; and lack of trained health care and laboratory workers.

**Conclusions:**

At this early stage in the global scale-up of testing for viral hepatitis, there is a wide variation in testing practices and approaches across different programmes. There remains limited access to NAT to confirm viraemia, and patient self-payment for testing and treatment is common. There was consensus from implementing organizations that scale-up of testing will require increased community awareness, health care worker training, development of national strategies and guidelines, and improved access to low cost NAT virological testing.

**Electronic supplementary material:**

The online version of this article (10.1186/s12879-017-2767-0) contains supplementary material, which is available to authorized users.

## Background

Hepatitis B virus (HBV) and C (HCV) infections are major causes of chronic liver disease and associated morbidity due to cirrhosis and hepatocellular carcinoma (HCC) globally and together accounted for an estimated 1.34 million deaths in 2015 [1]. The disease burden of chronic HCV and HBV infection is disproportionately high in low- and middle-income countries (LMICs), especially in East and South East Asia and in Sub-Saharan Africa for HBV infection [2]. However, despite the high prevalence of disease, and the availability of effective curative treatment for HCV infection using the new direct acting anti-viral (DAA) drugs, as well as long-term suppressive antiviral treatment for HBV, most people infected with HBV or HCV globally have never been tested and so remain unaware of their infection. Key reasons for this current very low rate of hepatitis testing in LMICs include: limited laboratory capacity and access to reliable, low-cost, HCV diagnostics, and lack of testing guidance specifically for LMICs [3].

WHO has recently developed guidelines on testing for hepatitis B and C infection that are intended as the basis for development of national guidelines for hepatitis testing in resource-limited settings [4]. Formulation of the recommendations was based on the GRADE approach (Grading of Recommendations, Assessment, Development and Evaluation) that include an assessment of the quality of evidence, but also considerations of patient and healthcare worker acceptability and preferences, resource use and feasibility [5, 6]. At present, although there are more than 40 published reports of different viral hepatitis testing programmes [7–55], the majority of these (88%) were from high-income countries, mainly the United States and Western Europe. In LMICs, there have been only two reports from Sub-Saharan Africa [27, 31], three from Asia [44, 49, 52] and one from Latin America [46]. We therefore undertook a survey of programmatic experience with testing for hepatitis B and C across a range of settings in LMICs, where access to laboratory infrastructure and specialised tests is limited. Our objective was to inform feasibility of potential recommendations on testing approaches (who and where to test) and how to test (selection of assays) in the WHO viral hepatitis testing guidelines, and also to assess key perceived barriers/challenges and strategies to address these and so guide implementation of hepatitis testing and treatment services.

## Methods

### Survey sites

Potential hepatitis testing and treatment programme sites in LMICs were identified from the various contacts databases of the WHO Global Hepatitis Programme (GHP) and of key WHO implementing partners [Médecins du Monde (MdM), Médecins sans Frontières (MsF) and Forum for Collaborative Research]. Twenty-two programmes from Asia, Sub-Saharan Africa and Eastern Europe that were broadly representative of different types of testing programmes ie. governmental or non-governmental organisations; hospital or community based testing; general or specific target populations; and from different geographic regions, were invited to participate in the survey.

### Survey questionnaire

A 33 question semi-structured questionnaire was developed by WHO and MdM, which was organized into five sections: PART A: Demographics of interviewees (professional profile, working experience); PART B: Programme information (Who is tested and where; what assays/algorithms are used; counselling and training; funding and costs of testing and treatment); PART C: Existence of protocol for viral hepatitis care and treatment; PART D: Perceived barriers/challenges and solutions; PART E: Provision of relevant epidemiological data. PARTS A to C comprised multiple choice standardized questions, with text fields to allow for additional comments, while PART D involved open-ended questions (Additional file 1). Interviews were conducted by telephone or in person by 7 persons (AI, NL, JB, SL, RC, NW, SH, PE) between June to September 2015, and immediately transcribed and sent to participants to ensure accuracy.

### Survey analysis

Questionnaire responses of PARTS A to C were analysed using descriptive statistics within Microsoft Excel. The written responses to PART D were analysed using a thematic analysis approach [56]. A systematic reading and coding of the transcripts allowed us to identify major themes and categories of perceived barriers and solutions for scale-up of testing, and six major categories were identified through consensus discussion within the study team.

## Results

### General characteristics

**Fig. 1:**
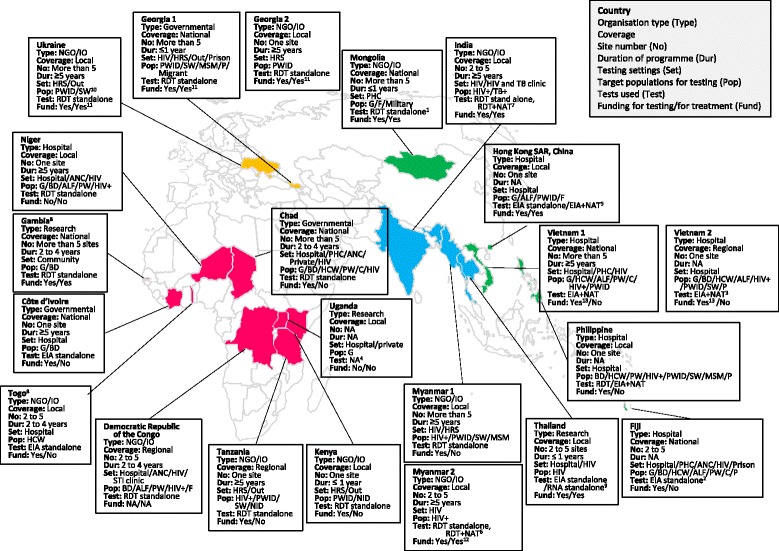
Geographic distribution and characteristics of the 22 testing programmes. *Categories of programme:* Governmental; NGO/IO (Non-governmental or international organization); Research; Hospital (hospital initiative). *Coverage of programme:* National; Regional; Local (only the population covered by the site). *Number of sites:* One; 2 to 5; > 5. *Duration of programme (year):* ≤1; 2 to 4; ≥5. *Testing settings:* Hospital; PHC (primary health care site); ANC (antenatal care site); HRS (harm reduction service); Out (outreach); HIV (HIV clinic); G (general population); BD (blood donor); PW (pregnant women); C (child); HCW (health care worker); HIV+ (HIV positive person); PWID (people who inject drugs); SW (sex worker); MSM (mem who have sex with men); P (Prisoner); F (family of HIV/HBV/HCV positive person); ALF (person with abnormal liver function test); STI (sexually transmitted infection); TB+ (tuberculosis positive person); NID (non-injecting drug user); NA (not answered). *Assays:* RDT (Rapid Diagnostic Test); EIA (Enzyme Immunoassay); NAT (Nucleic Acid Test). ^1^: Add NAT within 6 month after the RDT screening to confirm the chronic infection; ^2^: RDT was also available at the site; ^3^: Select test approach (RDT standalone, EIA standalone, RDT/EIA + NAT) based on the patient’s financial status; ^4^: RDT and NAT were available but test approach was not answered; ^5^: Apply NAT standalone to assess the eligibility of treatment; ^6^: Applied RDT + NAT standalone for HBV to assess the eligibility of treatment for children; ^7^: Apply RDT + NAT standalone for HCV limited to persons living in the city; ^8^: Offered test only for HBV; ^9^: EIA standalone for HBV and NAT standalone for HCV; ^10^: PWID for HCV and SW for HBV; ^11^: Financial support is available for HCV treatment but not available for HBV treatment; ^12^: The treatment for HBV-HIV co-infected person is covered by the programme. Financial support is available for HCV treatment; ^13^: Financial support is available for HBV testing but not for HCV testing

Overall, we evaluated 22 programmes from 19 countries [9 from the African region: Chad, Côte d’Ivoire, Democratic Republic of the Congo, Gambia, Kenya, Niger, Tanzania, Togo and Uganda]; 6 in Western Pacific: [Fiji, Hong Kong SAR/China, Mongolia, Philippines and Viet Nam (two programmes)]; 4 in South-East Asia: [India, Myanmar (two programmes) and Thailand]; and 3 in Eastern Europe: [Georgia and Ukraine (two programmes)]. All but four of the programmes were based in LMICs according to World Bank classification in 2015 [57]. Seven countries (36.8%) were classified as low-income countries (Chad, Democratic Republic of the Congo, Gambia, Niger, Tanzania, Togo and Uganda); eight (42.1%) as lower-middle income countries (Côte d’Ivoire, India, Kenya, Mongolia, Myanmar, Philippines, Ukraine and Viet Nam), and four (21.1%) as upper-middle or high-income countries (Fiji, Georgia, Hong Kong SAR/China and Thailand) (Table 1). The majority of these countries (12, 54.5%) had a high HBsAg prevalence in the general population and the main route of transmission was mother to child. In contrast, only four countries had a high HCV prevalence in the general population largely as a result of transmission through poor injection practices in the past, with an additional 6 countries with high prevalence in specific key populations such as people who inject drugs (PWID). The majority of the nine African countries had low prevalence (Table 2) [58–70]. Figure 1 shows the geographic distribution of these programmes annotated with key features (category of programme, level of coverage, number of sites, duration of programme, target population for testing, testing setting, type of test used, and availability of funding for testing and treatment).

**Table 1:** Characteristics of 22 viral hepatitis testing programmes

Programme characteristics	Total, *n* = 22 (%)
**Geographic location of testing programmes**	
Africa	9 (40.9)
Europe	3 (13.6)
South-East Asia	4 (18.2)
Western Pacific	6 (27.3)
**Income categories of countries where programmes conducted** ^a^ *n* = 19 countries	
Low-income	7/19 (36.8)
Lower-middle income	8/19 (42.1)
Upper-middle	3/19 (15.8)
High-income	1/19 (5.3)
**Programme coverage**	
National	7 (31.8)
Regional	3 (13.6)
Local	12 (54.5)
**Number of testing sites**	
More than five	7 (31.8)
Two to five	6 (27.3)
One	8 (36.4)
Not indicated	1 (4.5)
**Type of organisation leading programme**	
Non-governmental or international organization	10 (45.5)
Government	3 (13.6)
Hospital	6 (27.3)
Research institution	3 (13.6)
**Duration of programme**	
≥ 5 years	9 (40.9)
2 to 4 years	4 (18.2)
≤ 1 year	4 (18.2)
No response	5 (22.7)
**Target population and location of testing**	
Target population for testing	
Specific target populations only	11 (50)
General population^b^ only	2 (9.1)
General and specific target populations	9 (40.9)
Details of specific target population (multiple options possible)	
HIV positive	11 (50)
PWID	10 (45.5)
Clinical suspicion of hepatitis (Abnormal liver function tests or symptoms/signs)	6 (27.3)
Sex worker	6 (27.3)
Pregnant women	6 (27.3)
Health care worker	6 (27.3)
Prisoner	4 (18.2)
Family of HBV/HCV/HIV positive	3 (13.6)
Children of positive mothers	3 (13.6)
MSM	3 (13.6)
Other^c^	5 (22.7)
**Testing setting** (multiple options possible)	
Hospital-based	12 (54.5)
HIV clinic	10 (45.5)
Harm reduction service	6 (27.3)
Primary health care facility	4 (18.2)
Outreach programme	4 (18.2)
Antenatal clinic	4 (18.2)
Private sector	2 (9.1)
Community	1 (4.5)
Other^d^	4 (18.2)
**Approaches to testing**	
Who initiates testing? (multiple options possible)	
Provider	19 (86.4)
Client	8 (36.4)
Not indicated	2 (9.1)
Who delivers testing? (multiple options possible)	
Physician	11 (50)
Laboratory technician	5 (22.7)
Counsellor	5 (22.7)
Nurse	4 (18.2)
Other health care worker	4 (18.2)
Other^e^	3 (13.6)
Testing approach for HCV (20 programmes)^f^	
RDT standalone	12 (60, *n* = 20)
EIA standalone	4 (20, *n* = 20)
RDT/EIA + NAT	2 (30, *n* = 20)
NAT standalone	1 (5, *n* = 20)
Not indicated	1 (5, *n* = 20)
Testing approach for HBV (22 programmes)^g^	
RDT standalone	11 (50)
EIA standalone	6 (27.3)
RDT/EIA + NAT	4 (18.2)
Not indicated	1 (4.5)
Integrated testing (multiple options possible)	
With HIV	8 (36.4)
With HIV/HBV/HCV/Syphilis	6 (27.3)
With HBV/HCV	4 (18.2)
With HIV/HBV/HCV/TB	1 (4.5)
No integrated testing	5 (22.7)
Liver staging in those with positive test	
Not routinely done	6 (27.3)
Yes^h^	16 (72.7)
Counseling	
Pre−/post- counseling	15 (68.2)
No counseling/or unknown	7 (31.8)
**Access to treatment and funding**	
Treatment availability	
HBV^i^	18 (81.8)
HCV	14 (70, *n* = 20)
No treatment for either HBV and HCV	2 (10, *n* = 20)
Funding source for HCV testing (multiple options possible, 20 programmes)	
Support from NGO/IO/Government/Other donor	15 (75)
Patient self-payment	7 (35)
Private insurance	4 (20)
Funding source for HBV testing (multiple options possible, 22 programmes)	
Support from NGO/IO/Government/Other donor	19 (86.4)
Patient self-payment	8 (36.4)
Private insurance	4 (18.2)
Financial support for treatment	
For HBV	6 (27.3)
For HCV	7 (35, *n* = 20)

*PWID* people who inject drug, *MSM* men who have sex with men, *RDT* rapid diagnosed testing, *EIA* enzyme immunoassay, *NAT* nucleic acid testing, *HIV* human immunodeficiency virus, *HBV* hepatitis B virus, *HCV* hepatitis C virus, *TB* tuberculosis

^a^Based on the World Bank classification in 2015 [57]; ^b^General population included general population and blood donor; ^c^Other included non-injecting drug users, migrants, military and TB positive persons; ^d^Other included two prisons, one HIV/TB clinic and one sexually transmitted infection clinic; ^e^Other included self-testing; ^f^RDT/EIA + NAT (*n* = 3) as an optional approach; ^g^RDT/EIA + NAT (n = 2) as an optional approach to evaluate the treatment eligibility. One programme offered RDT standalone for blood donor screening; ^h^Fibroscan available (*n* = 9) and APRI score (*n* = 7); ^i^HBV treatment only available for HBV-HIV co-infected persons and not for HBV mono-infected persons (n = 9)

**Table 2:** Epidemic profiles of hepatitis B and C infection in the countries covered by the survey

**HBV epidemiology**	**Country number (%) and name**
High seroprevalence (>5%) in general population	12 (54.5%) Gambia, Thailand, Chad, Mongolia, Côte d’Ivoire, Vietnam, Niger, Uganda, Hong Kong SAR/China, Democratic Republic of the Congo, Tanzania, Kenya
Intermediate seroprevalence (3-5%) in general population	3 (13.6%) Fiji, Philippines, Myanmar
Low seroprevalence (<3%) in general population	3 (13.6%) Ukraine, India, Georgia
**HCV epidemiology based on anti-HCV antibody**	**Country number (%) and name**
High seroprevalence (>3%) in general population and concentrated epidemic among PWID	3 (13.6%) Georgia, Mongolia, Ukraine
Intermediate/low seroprevalence (1-3%) in general population but concentrated epidemic among PWID	5 (22.7%) India, Philippines, Thailand, Myanmar, Vietnam
Low seroprevalence (<1%) in general population	9 (40.9%) Chad, Côte d’Ivoire, Democratic Republic of the Congo, Gambia, Hong Kong SAR/China, Kenya, Niger, Tanzania, Uganda

*Source*: [58–70]; the HCV prevalence data provided by the respondents are presented for Côte d’Ivoire, Mongolia, Niger, Philippines, Togo and Uganda as no published data is available from these countries

Of the 22 programmes included in the analysis, 10 (45.5%) were implemented by non-governmental or international organizations (NGO/IO), namely MdM, MSF, Alliance Ukraine, and Expertise France; 3 (13.6%) through national governments; 6 (27.3%) by hospitals, and 3 by independent research institutions (13.6%). More than half of the programmes had been implemented for more than 2 years, and in 40.9% for longer than 5 years. Twelve of the programmes (54.5%) were implemented at just a single site, while 3 (13.6%) had a regional coverage, and 7 (31.8%) were being implemented nationally. Twenty programmes (90.9%) offered both HCV and HBV testing and two programmes in Gambia and Togo (9.1%) only offered testing services for HBV infection.

The majority (63.6%) of the 22 interviewees were medical doctors. Five (22.7%) of medical doctors worked as physicians within the programmes, 5 (22.7%) as programme managers and 4 (18.2%) as policy makers and implementers. The remaining 8 were non-medical workers of which 7 worked as programme coordinators. All interviewees had at least 3 years experience in the field and 5 (22.7%) had more than 10 years experience.

Overall, 94.7% of programmes were based in LMICs of which 7 countries (36.8%) were classified as low-income countries according to World Bank classification in 2015 [57] (Chad, Democratic Republic of the Congo, Gambia, Niger, Tanzania, Togo and Uganda). Eight countries (42.1%) were lower-middle income countries (Côte d’Ivoire, India, Kenya, Mongolia, Myanmar, Philippines, Ukraine and Viet Nam), and 4 (21.1%) were upper-middle and above income countries (Fiji, Georgia, Hong Kong SAR/China and Thailand).

### Setting and target population

The majority of programmes indicated that they offered hepatitis testing in hospital-based settings (54.5% of programmes), but specifically also in human immunodeficiency virus (HIV) clinics (45.5%), harm reduction services (27.3%), primary health care facilities (18.2%), and antenatal clinics (18.2%). Only four programmes (18.2%) undertook outreach (Georgia, Kenya, Tanzania and Ukraine) or only one (4.5%) undertook community-based testing (Gambia). All but two of the programmes included targeted testing of specific affected populations, especially people living with HIV (11, 50%) and PWID (10, 45.5%). There were only two programmes that were exclusively dedicated to general population testing (9.1%) (Côte d’Ivoire and Gambia). All NGO/IO supported programmes focussed on testing of key and marginalised populations and only one government programme (Chad) supported a general population testing approach (4.5%). The testing settings and target populations are described in Table 1 and Fig. 1.

### Diagnostic assays and testing algorithm

Most of the programmes (16, 77.3%) offered some form of integrated testing – combining HBV and HCV testing (11 of 17 programmes, 64.7%) or with HIV (15 of 17 programmes, 88.2%). The majority of hepatitis testing was provider-initiated by doctors, but in 8 (36.4%) programmes testing was initiated by the client (Chad, Côte d’Ivoire, Fiji, Georgia, Kenya, Myanmar, Togo and Ukraine). Very few programmes made use of community or peer workers.

Rapid diagnostic tests (RDT) alone were used respectively in 10 (50%) of the 20 programmes offering HCV testing, and 11 (50%) of 22 programmes offering HBV testing, and enzyme immunoassay (EIA) by 4 (20%) of 20 and 6 (27.3%) of 22 programmes, respectively. Nucleic acid testing (NAT) for confirmation of viraemia was available in only 6 (30%) of 20 programmes for HCV RNA (Hong Kong SAR/China, Mongolia, Thailand, Uganda and two programmes in Vietnam), and 4 programmes (18.2%) of 22 for HBV DNA (Myanmar, Philippine, Uganda and one programe in Vietnam). EIA and NAT were more likely to be available in hospital-based programmes.

Although, the majority (19, 86.4% for HBV and 15, 75% for HCV) of the programmes had a budget for testing, around a third of programmes required some patient self-payment for testing, and for 18.2% of programmes, this was partly covered by private insurance. In the case of treatment, around a third of programmes provided funding for HCV and HBV treatment, and two thirds required patient self-payment.

### Further investigations, care and treatment following diagnosis

Fifteen (68.2%) of the programmes offered pre- and/or post-counselling, and 16 (72.7%) offered some level of further evaluation and staging of liver disease using Fibroscan (9, 40.9%), or APRI or FIB-4 scoring based on readily available and cheap laboratory tests, such as liver transaminases and platelet count and other measures (7, 31.8%). Among the programmes providing HBV testing, only 12 (54.5%) offered HBV vaccination in those who tested negative.

Four and six programmes offered no access to treatment for HBV and HCV, respectively, despite offering testing. Although 18 programmes indicated that HBV treatment was available in the country, in 9 programmes this was only for HIV-HBV co-infected persons through use of tenofovir-based antiretroviral regimens, or for those able to self-pay. Among the programmes which conducted HCV testing, treatment was reported to be available in the country in 14 (70%) of the 20 programmes, but only for those able to self-pay, or as part of pilot programmes or clinical trials. The exception was Georgia where HCV treatment was available through the national programme in those with advanced liver disease.

### Qualitative evaluation of perceived barriers and strategies to scale-up viral hepatitis testing

Interviewees identified multiple different issues as barriers to testing, which were grouped into six key thematic categories: 1. Limited community awareness and education about viral hepatitis; 2. Lack of national guidance or policies and their implementation; 3. Funding – high costs of testing especially for NAT, and lack of dedicated funding for testing services; 4. Laboratory issues – poor infrastructure, poor quality tests and lack of quality assurance; 5. Service delivery and lack of trained healthcare workers to manage hepatitis; 6. Lack of availability of HBV treatment for mono-infected patients and to new DAAs for HCV treatment. Table 3 summarises the issues most commonly raised within these six categories as well as specific proposed interventions to address these barriers. The four most critical interventions identified were: awareness raising within the community; expanded access to assays, including point-of-care tests; access to low cost generic drugs; training and capacity building of laboratory staff and healthcare workers; and development of national guidelines.

**Table 3:** Key challenges in access to and scale-up of viral hepatitis testing and proposed interventions

Challenges (number of respondents highlighting issue)	Proposed interventions (number of respondents highlighting issue)
**1. Community awareness and education** *Lack of awareness among community* • **About disease and its consequences (9)** • About value and availability of testing services (1) *Health beliefs* • Delayed health-seeking behaviour especially in young men (2) • Self-treatment and traditional medicine (1) • Fear of stigmatization (1)	**1. Community awareness and education** • **Increase awareness (11)** • Increase implementation of HBV vaccination (2)
**2. Service delivery** *Laboratory infrastructure* **• Poor infrastructure and lack of staff (10)** • **Lack of access to NAT and FibroScan (8)** • Concern about low quality tests and lack of quality assurance (4) • Lack of supply management (3) • Lack of assay for hepatitis delta virus (1) • Dependence on blood sampling (1) • Distance to testing services (mainly in urban settings) (1) • Lack of laboratory network (1) *Poor linkage to hepatitis care* • **Lack of linkage to care (7)** • Lack of access to harm reduction services (2)	**2. Service delivery** **• Expand access to assays and technologies: eg. DBS, oral test, point of care test, self-testing, GeneXpert and Fibroscan (12)** • Establish good quality assurance on laboratory tests (6) • Decentralization of testing sites (3) • Establishment of referral pathway and expand laboratory networks, to include central/private laboratories and existing HIV testing infrastructure and existing HIV structure (3) • Offer integrated testing for multiple infections (for HCV, HBV, HIV) (2) • Expand work with key populations (1) • Prioritize testing in health care workers (1)
**3. Lack of access to treatment** • Unavailability of hepatitis treatments (11) • Lack of treatment for children (1) • Slow approval process for new medicines (1)	**3. Lack of access to treatment** **• Expand treatment availability and access to cheap generic medicine (7)**
**4. Health care workers (HCW)/laboratory education** *Lack of awareness among HCW and service providers* **• About disease and its consequences (6)** • About value and availability of testing services (3) • Lack of physicians who are able to treat hepatitis (especially in children) (2) *Lack of training* • **For HCW, laboratory technicians and physicians (6)**	**4. Health care workers (HCW)/laboratory education** **• Training/increase technical capacity of care teams in area of hepatitis (10)** • Need more staff (1) • Establish a degree in hepatitis research (1)
**5. National guidance and policies** • **Lack of national guidance (6)** • Policies not implemented (2) • No epidemiological data on viral hepatitis (2) • Policies are discriminating/stigmatizing (1)	**5. National guidance and policies** • **Development of national guidelines/strategy (7)** • Advocacy with policy makers (4) • Need surveillance data to identify settings and populations with high burden (3) • Development of policy on PMTCT for viral hepatitis (1)
**6. Funding** • **Lack of sustained funding commitment (9)** • High costs of testing and additional assays to determine treatment eligibility (3)	**6. Funding** • Development of funding strategy for testing and treatment (4) • Costing assessment across cascade of care (1)

*HCW* health care worker, *NAT* nucleic acid testing, *PMTCT* prevent mother to child transmission, *DBS* dried blood spot. Issues identified by more than five respondants are presented in bold

We also sought perspectives from interviewees on the ethics and rationale of testing by programmes where treatment was not available, given that there is still very limited access to HBV treatment for HBV mono-infected persons, and self-payment is currently required to access DAA HCV treatment for the majority of programmes. Key reasons given by respondents from 12 programmes for testing in the absence of treatment were to provide: (i) a platform for health education and health promotion; (ii) counselling of high risk anti-HCV negative persons on how to remain negative, or HBV vaccination of those who are HBV non-immune; and counselling of HCV and HBV positive persons and family members how to prevent transmission, and reduce disease progression, such as through alcohol cessation. Testing among health care workers for viral hepatitis was also identified as another situation where testing would be appropriate to enable vaccination of non-immune HCW even in the absence of treatment and adoption of measures to minimize risk of transmission from infected HCW to patients. Finally, testing was seen as an essential part of surveillance to gather national epidemiological data on HCV and HBV infection to inform advocacy for increasing treatment access and to raise awareness among the community.

## Discussion

There is still limited published experience of viral hepatitis testing and treatment in low- and middle-income countries to inform development of future programmes. This survey of 22 different hepatitis testing programmes in 19 predominantly low- and middle-income countries in Africa, Asia and Eastern Europe (of which 4 were from upper-middle income countries) representing a wide range of different HBV and HCV prevalence and epidemic patterns provides several valuable insights into current practices and future priorities in the delivery of hepatitis testing.

Overall, half of the programmes were being implemented by NGOs or IOs, and about half of them were only being implemented at a few sites, and so are not representative of national testing policies and approaches. The majority of testing programmes involved targeted testing of high-risk populations such as PWID, men who have sex with men (MSM), sex workers and prisoners, but also pregnant women and health care workers, alone or in combination with some general population testing approaches. NGO/IO supported programmes had a greater emphasis on testing of key and marginalised populations with the few government programmes supporting a general population testing approach. The majority of testing was undertaken in hospital-based settings, and in HIV, Tuberculosis (TB) and sexually transmitted infection (STI) clinics, and antenatal clinics. There was limited community-based or outreach testing. Of note, around half of the countries included in the survey have existing national policies on routine HBV screening for all pregnant women, and were Fiji, Georgia, Hong Kong SAR/China, India, Kenya, Myanmar, Philippine, Thailand and Ukraine [71–88].

There were several other common features to these testing programmes: First, two-thirds of the programmes were using a single RDT serological test for HBV and HCV. Second, 70% of the testing programmes were integrating viral hepatitis testing with existing clinics and services in HIV, TB and STI clinics as well as harm reduction services – alongside testing for HIV, TB and syphilis, as viral hepatitis is also prevalent in these populations. Third, in the programmes surveyed viral hepatitis testing was still provider-initiated by a physician in the majority of testing programmes, although this largely reflects practices in the predominantly facility-based programmes included in the survey. Fourth, staging of liver disease to assess eligibility for treatment was being undertaken by 73% of programmes, about a half of them with use of Fibroscan rather than the lower cost and more available APRI score. NAT testing to confirm the presence of viraemia and treatment eligibility was only performed by 6 of the HCV programmes and 4 of the HBV programmes. Fifth, it was of concern that 18% of programmes were not able to offer treatment for HBV mono-infection despite the wide availability of low cost generic tenofovir, and around a quarter were not providing HCV treatment. In addition, around a third of programmes were not routinely providing HBV vaccination, a low cost and highly effective preventative measure. Finally, although the testing costs were wholly or partly supported by the programme in more than 80% of the programmes, funding was covered by additional patient self-payment or private insurance in around a third. Treatment cost were covered by programmes in a third of cases.

The survey identified key barriers to testing services across different aspects of the health system but also some key strategies to address these challenges. Key strategies to facilitate access to testing identified from the survey were: awareness raising about viral hepatitis among the general population; decentralization of hepatitis testing with quality assurance on laboratory tests, and expanding access to technologies such as point of care NAT such as GeneXpert, dried blood spots sampling, use of oral RDT and self-testing; improving the training and capacity of staff in management of hepatitis; and incorporation of hepatitis testing and treatment into national health reimbursement programmes. Other diagnostic innovations to promote testing include multi-analyte testing, multiplex analysis or multi platforms for testing combined with syphilis [3, 89, 90]. Dried blood spot sampling may enhance access to both serological (using laboratory based EIA assays) and virological (using nucleic acid tests) testing [91]. However, at present, none of the manufacturers of commercial assays have validated their use with DBS samples or developed standard operating procedures, and nor is there any regulatory approval for their use from stringent regulatory authorities, such as WHO. The limited availability of low cost generic DAA treatments for HCV infection and tenofovir therapy for HBV mono-infected persons in many programmes was also identified as a key impediment to testing. Of note, 11 programmes highlighted the other benefits of testing in the absence of current access to treatment. These include opportunities to introduce measures and counselling to reduce transmission to family members and other close contacts including hepatitis B vaccination, and to counsel infected persons about measures to reduce disease progression of liver disease.

How well do our findings relate to existing reports on programmatic experience in viral hepatitis testing? We identified 49 published reports from different viral hepatitis testing programmes based on a PubMED search of existing relevant literatures on hepatitis B or C testing practices published since 2007 [7–55]. The majority of these (88%) were from high-income countries, mainly the United States and Western Europe, with only 6 from LMICs. These reports described testing programmes in a range of populations including: 13 of HCV birth-cohort testing in the context of the United States national recommendation to screen all adults living in the United States born between 1945 and 1965 at least once [8, 16, 19, 22–25, 38, 42, 45, 47, 51, 53]; 10 of HCV and one of both HBV and HCV in people who use drugs or harm reduction settings [10, 12, 20, 21, 32, 34, 35, 39–41, 51]; 4 of HCV in prisons [7, 15, 17, 54]; 3 of HBV testing in pregnant women [29, 46, 52]; and one of HBV and one of HCV in migrants [14, 43]. Of note, most of the programmes described were also community based or implemented in primary health care or prevention services. Although, descriptions of testing models and an evaluation of impact on cascade of care were provided in most of the reports, there was limited critical evaluation of programmatic lessons learnt.

In contrast, our survey was based on a broad range of facility and community based testing programmes from mainly LMICs with a focus on experiences with current testing practices, and common barriers. There were several key limitations to this survey. First, it was based on an opportunistic sample of 22 testing programmes that did not include any from Latin America or the Caribbean. Second, included programmes are not representative of national testing policies and approaches, as half of the programmes were being implemented by NGOs or IOs, and about half of them were only being implemented at a few sites. Third, the majority of programmes were also based in health facilities and there were few community based or outreach initiatives, which are likely to have different lab and service delivery approaches and unique challenges. There was also considerable variability in the way programmes were organised, especially between NGO/IO and governmental programmes, and so it was difficult to standardise reporting and data collection of their experiences, and to draw any inferences about programmatic effectiveness.

## Conclusions

What were the implications of our findings for both the formulation of recommendations in the WHO testing guidelines [4] and for future strategies to promote scale-up of viral hepatitis testing globally? The survey demonstrates that progress has been made in scale-up of hepatitis testing under the auspices of government and non-governmental initiatives, building on existing opportunities for testing through other services and on laboratory infrastructure. In spite of the many barriers highlighted, it also shows the feasibility of implementing HBV and HCV testing programmes across a wide range of LMICs. In particular, targeted testing is being effectively implemented among various higher risk groups, and RDTs have been widely adopted. However, there remains limited access to NAT for assessment of viraemia, and requirement for patient co-payment for testing and treatment remains a significant barrier.

These issues of feasibility and challenges encountered were considered in the formulation of recommendations on who to test and how to test in the 2017 WHO guidelines on testing for hepatitis B and C [4], with a promotion of the use of quality assured RDTs to promote access, strategies to promote linkage to care and treatment, use of simple low-cost non-invasive tests (NITs) such as APRI score for staging of liver disease, and universal adoption of targeted or focussed testing of specific populations most affected by HBV or HCV infection (i.e. who are either part of a population with higher seroprevalence or who have a history of exposure to or high-risk behaviours for HBV or HCV infection). These include HIV infected persons, PWID, prisoners, sexual partners and family members, including children of those affected by hepatitis B, pregnant women and health care workers. General population testing was recommended in high prevalence countries (above 5%) using existing testing infrastructure and approaches.

In terms of future policy, there is scope for increasing community-based testing and involving non-health workers with task-shifting to promote testing as achieved with HIV [92], and in access to point of care NAT technologies as well as dried blood spots sampling to improve access to virological testing. As countries progressively embrace universal health coverage, the current barrier to testing of costly self-payment of diagnostics can also be addressed. Finally, there is a need for more systematic reporting of experience with hepatitis testing and treatment programmes in LMICs. This will be further informed by a portfolio of important implementation science and demonstration projects supported by UNITAID and other organisations examining the impact of simplified and decentralised hepatitis C testing and treatment programmes.

## Additional file

Additional file 1: Questionnaire sheets for feasibility survey. (PDF 176 kb)

## Abbreviations

DAA: Direct acting anti-viral
EIA: Enzyme immunoassay
GHP: Global Hepatitis Programme
HBV: Hepatitis B virus
HCC: Hepatocellular carcinoma
HCV: Hepatitis C virus
HIV: Human immunedeficiency virus
IO: International organizations
LMICs: Low- and middle-income countries
MdM: Médecins du Monde
MSF: Médecins sans Frontières
MSM: Men who have sex with men
NAT: Nucleic acid testing
NGO: Non-governmental organizations
PWID: People who inject drugs
RDT: Rapid diagnostic tests
