# Supplementary material for: Survey of programmatic experiences and challenges in delivery of hepatitis B and C testing in low- and middle-income countries
Source: BMC Infect Dis. 2017 Nov 1;17(Suppl 1):696. doi: 10.1186/s12879-017-2767-0 (PMC5688462; doi:10.1186/s12879-017-2767-0)
Supplement: Additional file 1: — Questionnaire sheets for feasibility survey. (PDF 176 kb) [file 12879_2017_2767_MOESM1_ESM.pdf]

FEASIBILITY SURVEY

The purpose of this brief questionnaire is to inform the forthcoming WHO hepatitis B and C testing guidelines meeting at the end of September and planned WHO testing guidelines.

We are seeking to gather experiences of hepatitis B and/or C testing from as many different settings and populations in low and middle income countries. The settings include primary care, antenatal clinics, drug dependency services, HIV clinics, and populations – MSMs, injecting drug users, prisoners, pregnant women, HIV positive persons.

The questionnaire has four main components:
PART A: Background demographics of interviewee
PART B: Details of programme, including xxxx
PART C: Protocol for hepatitis care and treatment
PART D: Summary of perceived barriers and challenges, and potential solutions/strategies
PART E: Provision of relevant epidemiological data

The questionnaire will be interviewer administered and take approximately 30 to 40 minutes to complete.

We appreciate your time to complete this survey.

PART A - DETAIL OF INTERVIEW

INTERVIEWER

Date of completion dd /mm / 2015 ____

Name of interviewer ____

Contact (Tel, email) ____

RESPONDANT

Name ____

Professional profile (tick all that apply)

- Medical doctor /clinical officer ☐
- Medical assistant ☐
- Laboratory expert ☐
- National program administrator ☐
- Employee of an international organization (e.g., WHO) ☐
- Researcher ☐
- Employee/Consultant of a national or international NGO ☐
- Primary care provider ☐
- Programme implementer ☐
- Policy maker ☐
- Other ☐

Contact (Tel, email) ____

Organisation ____

City ____

Country ____

Your role in hepatitis testing program ____

specify/give details , ____

Working experience in Hepatitis testing program

- Less than 1 year ☐
- 1-2 years ☐
- 3-5 years ☐
- 5-10 years ☐
- More than 10 years ☐

PART B - DETAILS OF PROGRAMME

B1 GENERAL INFORMATION

B1.1 Do you do HCV, HBV, or both testing at your site?

	HCV	HBV
Yes	☐	☐
No	☐	☐

B1.2 Give a brief description of your programme:
National context, date of beginning, principal and specific objectives, main activities

B1.3 Country ____

B1.4 Site (tick all that apply)

- National level ☐
- Regional level (Province or State) ☐
- Site-specific (only at the program you work) ☐
- Other ☐ specify/give details , ____

B1.5 How many site are you working for?

- 1 ☐
- 2-5 ☐
- More than 5 ☐

B1.6 When did the testing activity start? dd /mm / yyyy

B2 DESCRIPTION OF HEPATITIS TESTING SERVICE

B2.1 Where is the testing is done?
(tick all that apply)

	HCV	HBV
Primary health care	☐	☐
Obstetric service	☐	☐
HIV service	☐	☐
Harm reduction service	☐	☐
Outreach	☐	☐
Prison	☐	☐
Hospital	☐	☐
Specialized center	☐	☐
Other	☐	☐ specify/give details, ________

B2.2 Who are the target populations for hepatitis screening in your programme?

	HCV	HBV
General population	☐	☐
Pregnant women	☐	☐
Children	☐	☐
Person who have chronic ill	☐	☐
Blood donors	☐	☐
Health care workers	☐	☐
Migrant	☐	☐
Injecting drug user	☐	☐
Sex worker	☐	☐
Men having sex with men	☐	☐
Prisoners	☐	☐
HIV positive people	☐	☐
Other	☐	☐ specify/give details, ________

B2.3 Is there integrated testing with other diseases?

Yes for HCV ☐ specify/give details, ________
Yes for HBV ☐ specify/give details, ________
No ☐

B2.4 Is there dual testing (HBV+HCV)

Yes ☐
No ☐

B2.5 Specify / give details on indication/criteria for testing.
For instance:
First antenatal visit, ART initiation, immunization, secondary prevention, national policies, national treatment program community mobilization through demand creation etc.

HCV ☐

HBV ☐

B2.6 What assays are used for HCV and HBV testing at your programme?
(tick all that apply)

HCV		Please give details of the testing kit names
RDT (rapid diagnostic test)	☐	________
EIA (Enzyme immunoassay)	☐	________
HCV core antigen test (HCV cAg)	☐	________
HCV RNA test	☐	________
Not sure	☐	

HBV		Please give details of the testing kit names
RDT (rapid diagnostic test)	☐	
EIA (Enzyme immunoassay)	☐	
HBV DNA test	☐	________
Not sure	☐	________

B2.7 What testing algorithm is used at your programme?

HCV	
One test strategy: RDT	☐
One test strategy: EIA	☐
One test strategy: HCV core Ag	☐
One test strategy: HCV RNA	☐
Two tests strategy: RDT/EIA + HCV cAg	☐
Two test strategy: RDT/EIA + HCV RNA	☐
Others	☐ specify/give details, ________
Not sure	☐

HBV	
One test strategy: RDT	☐
One test strategy: EIA	☐
One test strategy: HBV DNA	☐
Two test strategy: RDT/EIA + HBV DNA	☐
Others	☐ specify/give details, ________
Not sure	☐

B2.8 Which test is used for liver assessment?

	HCV	HBV
Not routinely done	☐	☐
APRI	☐	☐
Fib4	☐	☐
Other biomarkers	☐	☐
Fibro scan	☐	☐
Liver biopsy	☐	☐

Specify other biomarkers ☐

B2.9 Who delivers the testing pathway?
(tick all that apply)

	HCV	HBV
Counsellor	☐	☐
Physician	☐	☐
Laboratory technician	☐	☐
Nurse	☐	☐
Midwife	☐	☐
Health care worker	☐	☐
Other	☐	☐

specify/give details, ______________________

B2.10 Is the testing provider- or client-initiated?

	HCV	HBV
Provider initiated	☐	☐
Client initiated	☐	☐

B2.11.1 Which counselling and information is provided? (tick all that apply)

	HCV	HBV
No counselling	☐	☐
Pre-test counselling	☐	☐
Post-test counselling	☐	☐
Not Sure	☐	☐

If Counselling is provided, please answer the following quest ion. If Not, please skip to B3

B2.11.2. Is specific guidance/document available for the person in charge of testing?

	HCV	HBV
Yes	☐	☐
No	☐	☐

B2.11.3 What training have these persons received

Briefly describe here the modality of training (individual vs. collective - formal vs. mentoring - # of days)

B3 COSTS, BUDGET AND FUNDING

B3.1 Who pays for testing?

	HCV	HBV
Patient (self-payment)	☐	☐
Government (Public sector)	☐	☐
Private insurance	☐	☐
NGO	☐	☐
Other	☐	☐

specify/give details, ______________________

B3.2.1 Do you have an allocated budget for this testing and/or treatment programme for HCV/HBV?

	HCV	HBV
For testing Yes	☐	☐
No	☐	☐

	HCV	HBV
For treatment Yes	☐	☐
No	☐	☐

If yes, please answer B3.2.2, if not please answer B3.2.3

B3.2.2. What is the source of funding?

HCV

HBV

B3.3 Please provide estimated costs per year for the following.
If exact costs are unknown please provide estimates or proportions of overall costs

HCV Testing		Currency
Personnel	Personnel costs	
	Training	
Capital	Transport	
	Equipment	
	Infrastructure	
Recurrent	Test kits	
	Other	

HBV Testing		Currency
Personnel	Personnel costs	
	Training	
Capital	Transport	
	Equipment	
	Infrastructure	
Recurrent	Test kits	
	Other	

PART C - FOLLOW UP AND LINKAGE TO CARE

C.1 What is the protocol for care and management in the following :

People HCV Ab positive (with or without RNA positive)

	Yes	No
Specific counselling	☐	☐
Secondary investigations	☐	☐
Specific medical follow up	☐	☐
ARV for those HIV+	☐	☐
Treatment	☐	☐
Patients group	☐	☐
Offer HBV test	☐	☐
HBV immunization	☐	☐
Other	☐	☐

specify/give details, ____________________

Brief description (type of investigation or follow-up / setting / funding, etc.)

People HCV Ab negative

	Yes	No
Offer HBV test	☐	☐
HBV immunization	☐	☐

Describe the conditions (setting, funding, etc.)

People HB surface antigen (HBsAg) positive

	Yes	No
Specific counselling	☐	☐
Secondary investigations	☐	☐
Specific medical follow up	☐	☐
ARV for those HIV+	☐	☐
Treatment	☐	☐
Patients group	☐	☐
Other	☐	☐

specify/give details, ____________________

Brief description (type of investigation or follow-up / setting / funding, etc.)

People HBsAg Negative

	Yes	No
Offer HBV immunization	☐	☐

Describe the conditions (setting, funding, etc.)

C.2 Where is treatment provided?

- Same site ☐
- Different site ☐ specify/give details, ____________________
- Treatmnet not available ☐

C.3 How are you approaching testing in the absence of treatment?

PART D - BARRIERS AND CHALLENGES

D.1. What are the main barriers you have identified for testing implementation (at provider, patient and facility level) and what are your suggestions to facilitate access and uptake of testing and linkage to care.

Barriers	Suggestions
Common for HCV and HBV<br>Health system perspective<br>*Lack of national policies, guidelines, funding etc.*	*For instance: To not skip the training of health professionals, To organize awareness rising in the targeted community etc.*
Service provider perspective<br>*Lack of awareness of health professionals, overwhelmed consultation, Lack of counselling services, Accessibility to centralized lab and specialist*	
Technology perspective<br>*Lack of trust in testing procedures, Lack of quality assurance, Lack of laboratory staff capacity, Lack of testing kits, Lack of infrastructures*	
Patient perspective<br>*Lack of awareness of patients, fear / misconceptions about the disease, affordability for testing if self-pay*	
Others	
Specific for HCV	
Specific for HBV	

D.2 What are the most important components of international guidelines to facilitate the implementation of hepatitis testing as part as a health program in resource limited setting?

E1. Country prevalence in the general population and/or risk groups, available.

**HCV**	**HBV**
Source of the information, if known	Source of the information, if known

E2. Do you have information on your testing programme?

	HCV	HBV
Yes	☐	☐
No	☐	☐

If yes, please provide how many tested in different risk groups, prevalence and how many of risk in these groups for future testing your setting. (breakdown by risk groups and by gender, if possible)

Please provide information in spread sheet if preferred.

HCV Year of investigation

Specific Groups	Number of tested	Number of positive (%)	Potential Number at risk

HBV Year of investigation

Specific Groups	Number of tested	Number of positive (%)	Potential Number at risk
